# Gunao-Yizhi decoction combined with donepezil for vascular dementia: A systematic review and meta-analysis

**DOI:** 10.1097/MD.0000000000030971

**Published:** 2022-10-07

**Authors:** Yibin Hu, Lijuan Zhang, Xiuju Guan, Hanru Hou, Shuyue Bi, Changning Liu, Mingxiang Li, Kangfeng Wang

**Affiliations:** a College of Traditional Chinese Medicine, Shandong University of Traditional Chinese Medicine, Jinan, Shandong Province, People’s Republic of China; b Clinical Education Management Division, Affiliated Hospital of Shandong University of Traditional Chinese Medicine, Jinan, Shandong Province, People’s Republic of China; c First College of Clinical Medicine, Shandong University of Traditional Chinese Medicine, Jinan, Shandong Province, People’s Republic of China; d Department of Neurology, Affiliated Hospital of Shandong University of Traditional Chinese Medicine, Jinan, Shandong Province, People’s Republic of China.

**Keywords:** donepezil, Gunao-Yizhi decoction, meta-analysis, vascular dementia

## Abstract

Gunao-Yizhi decoction has the effects of supplementing intelligence, strengthening marrow, resolving phlegm, and reducing turbidity. It is clinically used for the treatment of vascular dementia (VaD). However, there is still a lack of systematic evaluation of its efficacy and safety. This review conducted a systematic review of the current evidence on the efficacy and safety of Gunao-Yizhi decoction combined with donepezil for VaD.

**Methods:**

China National Knowledge Infrastructure (CNKI), Wanfang database (Wanfang), Chinese Science and Technology Periodical Database (VIP), China Biology Medicine disc (CBM), MEDLINE, EMBASE, and Cochrane Library were searched for randomized controlled trials on Gunao-Yizhi decoction combined with donepezil for VaD. RevMan 5.3 software was used for data analysis.

**Results:**

Twelve studies were obtained, including 1036 patients. Compared with donepezil alone, meta-analysis showed that Gunao-Yizhi decoction combined with donepezil could improve clinical efficacy, mini-mental state examination (MMSE) score, Hasegawa dementia scale (HDS), increase the level of superoxide dismutase (SOD) in serum, and reduce the level of malonaldehyde dismutas (MDA) in serum. The GRADE system was adopted to evaluate the outcome index. Clinical efficiency and the MMSE score were evaluated as very-low-quality evidence. HDS score, serum SOD level, and serum MDA level were evaluated as low-quality evidence.

**Conclusion:**

Gunao-Yizhi decoction combined with donepezil has a significant prevalence in the treatment of vascular dementia, with no increase in adverse events. Gunao-Yizhi decoction can be recommended for routine use in the treatment of VaD.

## 1. Introduction

Vascular dementia (VaD) is a serious manifestation of vascular cognitive impairment (VCI), accounting for 12 to 20% of all causes of dementia, only inferior to Alzheimer disease (AD). It can be secondary to stroke and other cerebrovascular diseases.^[1]^ Research shows that the prevalence of VaD in the elderly in China increases year by year.^[2]^ VaD has a significant impact on patients’ mental state and self-care ability, which causes a severe economic burden to patients and their families and causes enormous medical pressure on the country.^[3]^ At present, there is no specific cure for VaD. The best-studied treatments are cholinesterase inhibitors and memantine, both licensed and well-established drugs for Alzheimer disease, albeit with modest effectiveness.^[4]^ Traditional Chinese medicine plays a vital role in preventing and treating VaD, and it has been widely used in clinics.

Gunao-Yizhi decoction is a decoction for treating vascular dementia by tonifying kidney and removing phlegm. Its specific prescription includes *12g Rehmannia glutinosa (Shudi), 12g Fructus Corn i(Shanyurou), 12g Lycium barbarum (Gouqi), 12g Radix Polygoni Multiflori Preparata (Zhishouwu), 12g Ligustrum Lucidum (Nvzhenzi), 9g Rhodiola rRosea (Hongjingtian), 9g Epimedium brevicornum (Yinyanghuo), 12g Semen Ziziphi Spinosae (Suanzaoren), 9g Acorus tatarinowii Schott (Shichangpu), Semen Astragali Complanati (Shayuanzi), 12g Salvia miltiorrhiza (Danshen), 9g Polygala tenuifolia (Yuanzhi), 18g Codonopsis pilosula (Dangshen), 12g Pueraria lobate (Gegen), 9g Rhizoma Chuanxiong (Chuanxiong), 9g Lumbricus (Dilong), 12g semen persica e(Taoren) ,12g Caulis Piperis Kadsurae (Haifengteng*). The above herbs are mixed with water, cooked until 400 mL of residual volume, and divided into 2 equal parts in terms of usage and dosage. Patients are suggested to take the warm mixture twice a day, once in the morning and once in the evening. Although Gunao-Yizhi decoction is widely used in the treatment of VaD, there has yet to be an objective evaluation of its clinical efficacy and safety. As a result, this paper will use a Cochrane systematic review to assess the efficacy of Gunao-Yizhi decoction and its modified prescriptions in combination with donepezil in the treatment of vascular dementia in order to provide an evidence-based basis for preventing and treating vascular dementia with traditional Chinese medicine.

## 2. Data and Methods

### 2.1. Study registration

The protocol for this systematic review was registered on INPLASY and the registration number is INPLASY202240094 (URL= https://inplasy.com/inplasy-2022-4-0094/).

### 2.2. Research data

All randomized control trials of Gunao-Yizhi decoction combined with donepezil for VaD were included. Trials in English and Chinese were included.

### 2.3. Research methods

#### 2.3.1. Literature inclusion criteria

Randomized controlled trials (RCTs) on VaD were included, including English and Chinese literature. Patients who are diagnosed with vascular dementia according to the *Clinical Diagnostic Criteria of Vascular Dementia*,^[5]^ *Diagnostic Efficacy Criteria of TCM Diseases*,^[6]^ or other diagnostic criteria for VaD. There was no specific restriction on age, gender, nationality, and race. The experimental groups were treated with Gunao-Yizhi decoction combined with donepezil, while the control groups were treated with donepezil alone.

#### 2.3.2. Literature retrieval criteria

We reviewed databases including China National Knowledge Infrastructure (CNKI), Chinese Science and Technology Periodical Database (VIP), Wanfang database (Wanfang), China Biology Medicine disc (CBM), PubMed, MEDLINE, and Cochrane library. The search date for the system was built to April 1, 2022. We used keyword combinations to retrieve literature, such as Gunao-Yizhi decoction OR Gunao-Yizhi OR vascular dementia OR vascular cognitive impairment OR post-stroke dementia OR post-stroke cognitive impairment OR dementia OR VD OR VaD.

#### 2.3.3. Literature exclusion criteria

Duplicate literature. Non-Chinese and non-English literature. Reviews. Non-human tests such as animal or cell tests. Medical experience summaries, case studies, and so on. RCTs that used other traditional Chinese medicine treatment methods, such as acupuncture, moxibustion, and others, may interfere with test results in addition to oral drugs.

#### 2.3.4. Data extraction and quality assessment

Two researchers extracted the data independently and assessed the quality of the included trials. The literature’s inclusion and exclusion criteria were strictly followed. If 2 researchers disagreed on the selection of a literature, the literature would be given to a third researcher who would analyze it and decide whether or not to keep it. To assess the quality of evidence in each comparison, we used the grading of recommendations assessment, development, and evaluation (GRADE) system. The risk of publication, heterogeneity, indirectness, imprecision, and publication bias were all evaluated, and the results were classified as high, moderate, low, or very low.

#### 2.3.5. Risk of bias assessment

Two researchers independently used the Cochrane risk-of-bias tool to methodological quality of the included trials. If there was insufficient data, we would try to contact the authors. If the data was missing, we would exclude the study.

#### 2.3.6. Statistical analysis

We used RevMan 5.3 software for meta-analysis and heterogeneity test of literature. I square test was used to detect the heterogeneity among included trials. The trials with significant heterogeneity (*P* < .10, I^2^ > 50%) were analyzed by random effects model; For the trials with small heterogeneity (*P* > .10, I^2^ < 50%), the fixed effects model was used for analysis. The number of studies in each comparison was small, so there was no subgroup analysis performed. We used the method of excluding each study one by one for the sensitivity analysis.

### 2.4. Outcomes

Total effective rate, mini-mental status examination (MMSE) score are the main outcomes.

Hasegawa dementia scale (HDS) score, the level of serum superoxide dismutase (SOD), and the level of serum malonaldehyde dismutase (MDA) are the secondary outcomes.

In each included study, we also recorded adverse events to see if Gunao-Yizhi decoction increased the risk of adverse events.

### 2.5. Ethical approval

This study does not require ethical approval because it is based on previously published research.

## 3. Results

### 3.1. Literature search results

**Figure 1.**
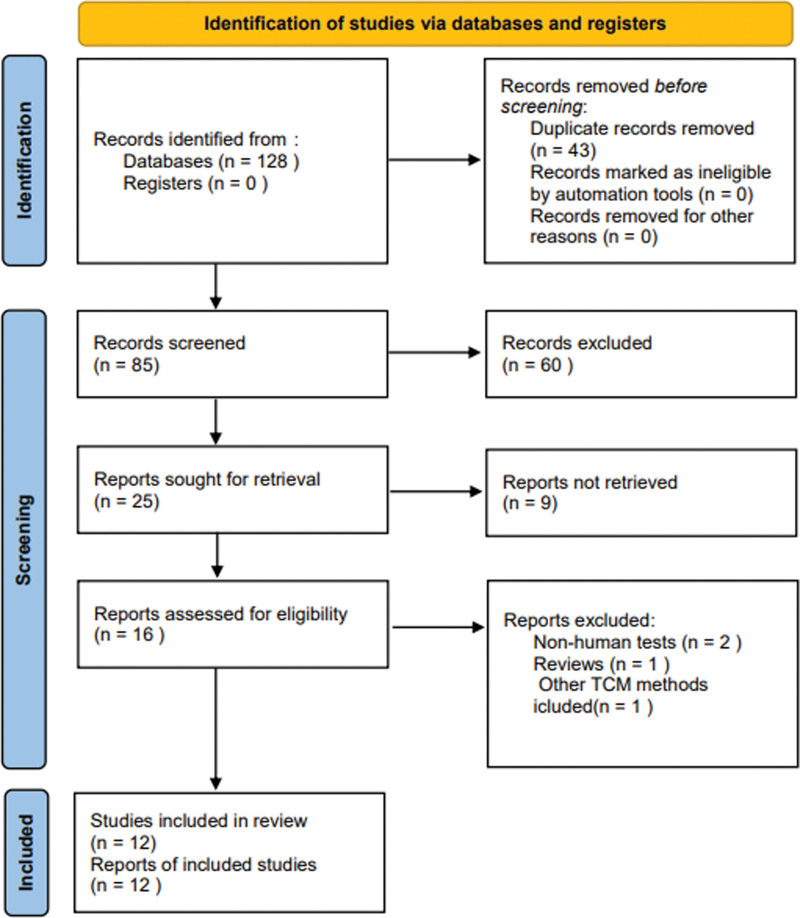
PRISMA flow chart.

According to the search strategy, 128 studies were identified from the databases. We imported the studies into EndnoteX9 software, and 43 duplicate studies were removed, 60 studies such as animal experiments, medical reviews, and case reports were removed after reading the abstract and title, and 12 studies were finally included after reading the full text according to the inclusion and exclusion criteria. The PRISMA flow chart is shown in Figure 1.

### 3.2. Summary of the included studies

Finally, 12 studies with a total of 1036 patients were included. All of the studies included were in Chinese. All patients in the experimental group received Gunao-Yizhi decoction in combination with donepezil, while patients in the control group received donepezil alone. Summary of the included studies is shown in Table 1.

**Table 1:** Summary of included studies.

Literature source	Sample size (experimental group/control group	Intervention measures (experimental group/control group)	Intervention time	Clinical outcomes
Gao Lu^[7]^ 2018	120 (60/60)	Gunao-Yizhi decoction + Donepezil/Donepezil	3 mo	②③④⑤
Li Yanyan^[8]^ 2021	60 (30/30)	Gunao-Yizhi decoction + Donepezil/Donepezil	3 mo	④⑤
Li Yang^[9]^ 2019	90 (45/45)	Gunao-Yizhi decoction + Donepezil/Donepezil	3 mo	②③
Liu Feng^[10]^ 2019	80 (40/40)	Gunao-Yizhi decoction + Donepezil/Donepezil	1 mo	①
Liu Yixiang^[11]^ 2017	55 (28/27)	Gunao-Yizhi decoction + Donepezil/Donepezil	3 mo	①③
Su Yingxia^[12]^2017	68 (34/34)	Gunao-Yizhi decoction + Donepezil/Donepezil	3 mo	③
Sui Xiaofeng^[13]^ 2017	100 (50/50)	Gunao-Yizhi decoction + Donepezil/Donepezil	3 mo	③
Sun Wei^[14]^ 2015	86 (43/43)	Gunao-Yizhi decoction + Donepezil/Donepezil	3 mo	③④⑤
Wang Zheng^[15]^ 2019	90 (45/45)	Gunao-Yizhi decoction + Donepezil/Donepezil	3 mo	③④⑤
Xue Lihong^[16]^ 2021	72 (36/36)	Gunao-Yizhi decoction + Donepezil/Donepezil	3 mo	②
Yang Shijie^[17]^ 2017	115 (60/55)	Gunao-Yizhi decoction + Donepezil/Donepezil	1 mo	④⑤
Yuan Jie^[18]^ 2017	100 (50/50)	Gunao-Yizhi decoction + Donepezil/Donepezil	3 mo	④⑤^1^

①Clinical efficiency; ②MMSE score; ③HDS score; ④Serum SOD Level; ⑤Serum MDA level.

HDS = Hasegawa dementia scale, MDA = malonaldehyde dismutase, MMSE = mini-mental status examination, SOD = superoxide dismutase.

### 3.3. Assessment of risk of bias

**Figure 2.**
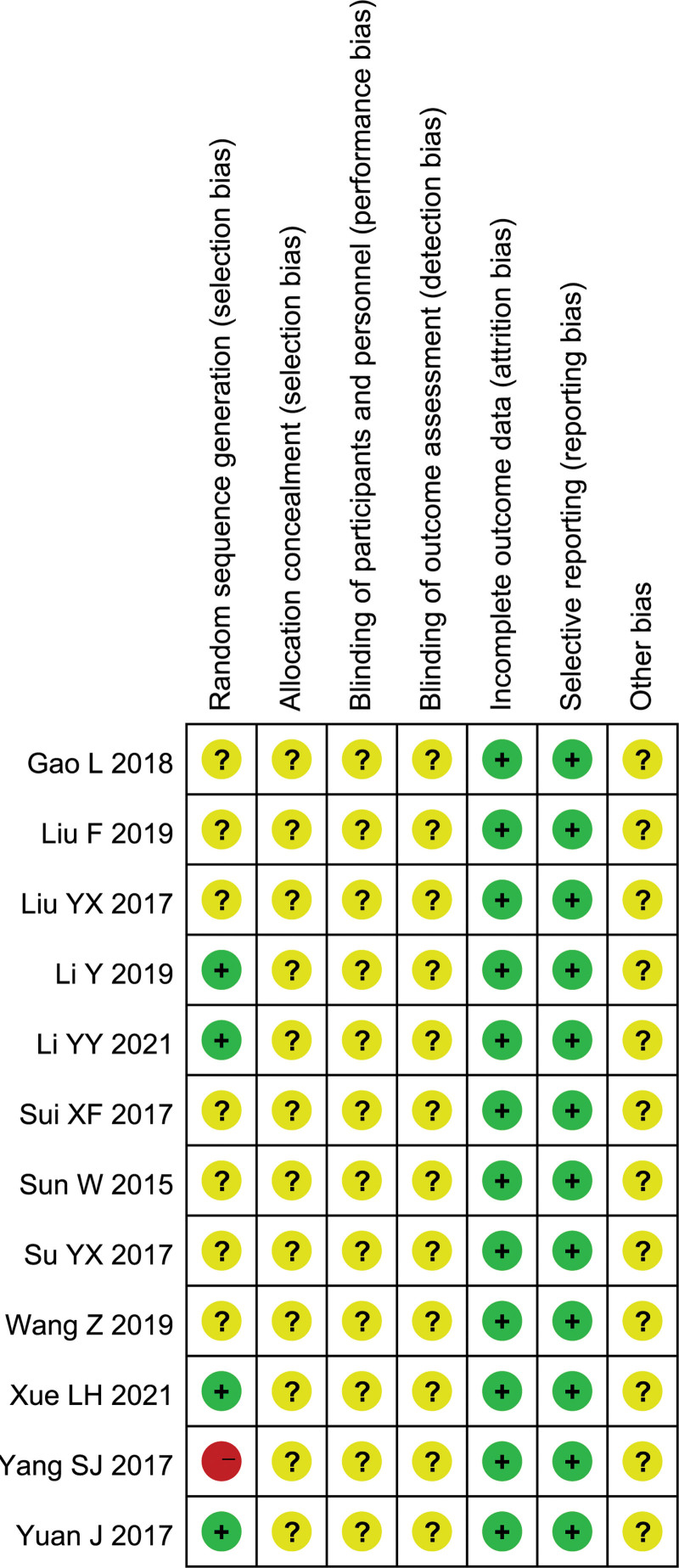
Risk of bias graph.

Among the 12 studies included, only 3 trials adopted a random-number table, so these were classified as low risk,^[8,9,16]^ 7 trials only mentioned random grouping without specifying the specific method, and these were considered as unclear risk,^[7]^ 1 trial only explained grouping according to the treatment method, which was considered as high risk.^[17]^ Because none of the studies mentioned allocation concealment or blinding, these were all regarded as unknown risk. There was no selective reporting in any of the studies, and it was unclear whether there was any other bias. The risk of bias in the included studies is shown in Figure 2.

### 3.4. Meta-analysis results

#### 3.4.1. Total effective rate

**Figure 3.**
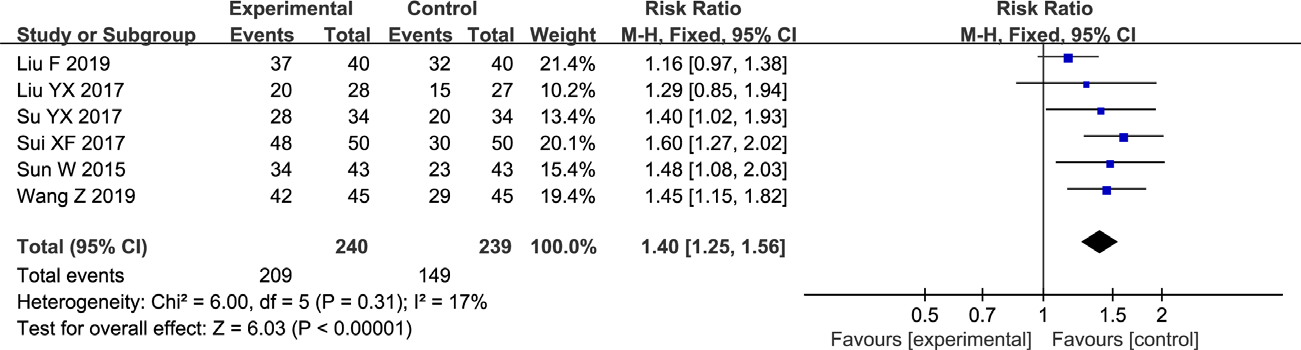
Forest plot of total effective rate.

Total effective rate is the sum of “significant effective rate” and “effective rate”. Six trials reported total effective rate (the criteria for judging the curative effect includes TCM syndrome standard and MMSE scoring standard), with a total of 479 patients. The heterogeneity test showed that I^2^ = 17%. The combined analysis of the fixed effects model showed that the difference in effective rate was statistically significant (RR = 1.40, 95% Cl [1.25, 1.56], *P* < .00001), indicating that clinical efficiency of Gunao-Yizhi decoction combined with donepezil was higher than that of donepezil alone. The results are shown in Figure 3.

#### 3.4.2. MMSE score

**Figure 4.**
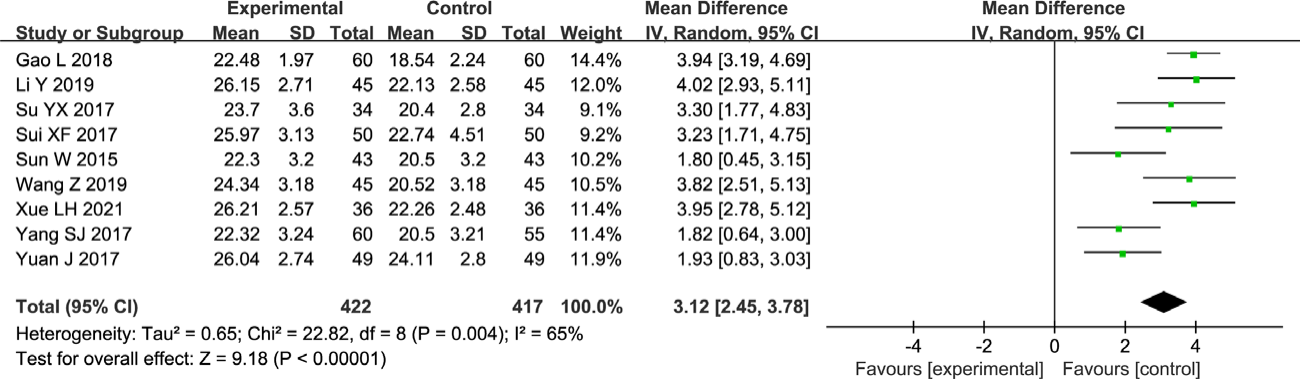
Forest plot of MMSE score.

Nine trials reported MMSE scores, with a total of 839 patients.^[7,9]^ There is significant heterogeneity among the included studies (*P* = .004, I^2^ = 65%). Sensitivity analysis revealed that excluding any study did not significantly reduce heterogeneity, which could be attributed to the control group’s intervention measures and the patients’ age. Gunao-Yizhi decoction combined with donepezil was better than donepezil alone in improving the MMSE scores of patients with vascular dementia (MD = 3.12, 95% CI [2.45,3.78], *P* < .00001). The results are shown in Figure 4.

MMSE = mini-mental status examination.

#### 3.4.3. HDS score

**Figure 5.**
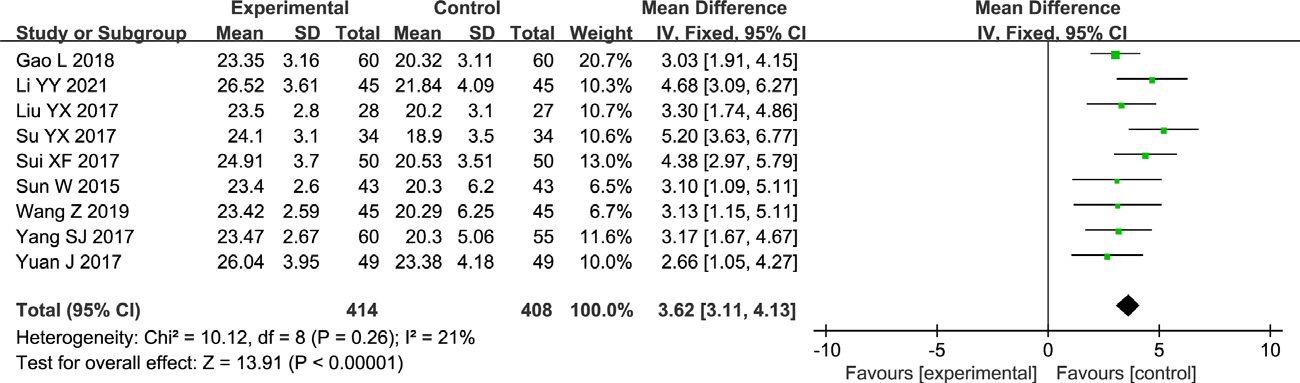
Forest plot of serum HDS level.

Nine studies reported HDS scores with a total of 822 patients.^[7,9]^ The heterogeneity test showed that *I*^2^ = 21%, which was combined and analyzed by fixed effects model. The results of the meta-analysis showed that Gunao-Yizhi decoction combined with donepezil was better than donepezil alone in improving the HDS score of patients with vascular dementia (MD = 3.62,95% CI [3.11,4.13], *P* < .00001). The results are shown in Figure 5.

HDS = Hasegawa dementia scale.

#### 3.4.4. Serum SOD level

**Figure 6.**
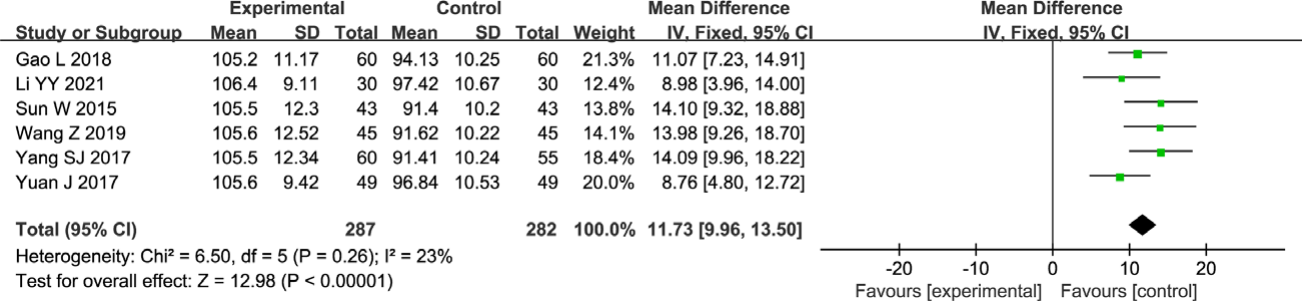
Forest plot of serum SOD level.

Six studies reported SOD level, with a total of 569 patients.^[7,8,14,15,17,18]^ The heterogeneity test showed that *I*^2^ = 23%, which meant there was no significant heterogeneity among the studies, so fixed effects model was adopted. Meta-analysis showed that the effect of Gunao-Yizhi decoction combined with donepezil on improving serum SOD levels in patients with vascular dementia (MD = 11.73, 95% Cl [9.96, 13.50], *P* < .00001) was better than that of donepezil alone. The results are shown in Figure 6.

SOD = superoxide dismutase.

#### 3.4.5. Serum MDA level

**Figure 7.**
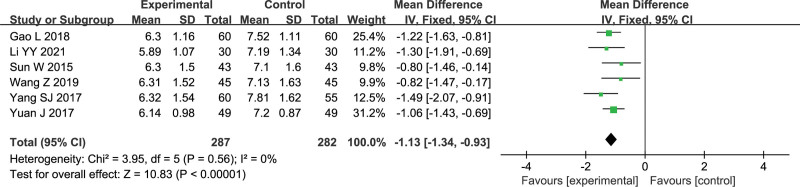
Forest plot of serum MDA level.

Six studies reported serum MDA level, with a total of 569 patients.^[7,8,14,15,17,18]^ The heterogeneity test showed *I*^2^ = 0%, indicating no heterogeneity between groups. So fixed effects model was adopted. Meta-analysis showed that the effect of Gunao-Yizhi decoction combined with donepezil on reducing MDA levels in patients with vascular dementia (MD = –1.13, 95% Cl [–1.34, –0.93], *P* < .00001) was significantly better than that of donepezil alone. The results are shown in Figure 7.

MDA = malonaldehyde dismutase.

### 3.5. Adverse events

One study described patients’ adverse events after taking medicine, including 2 cases of stomach discomfort and 1 case of mild drowsiness in the experimental group; There were 2 cases of mild drowsiness in the control group, which did not cause serious adverse consequences.^[18]^ Other studies did not describe the occurrence of adverse events.

### 3.6. Quality of evidence

We applied the GRADE system to assess the quality of evidence for each outcome.^[19]^ The results suggested that four main factors reduce the quality of evidence: All the studies did not mention the blinding method or the specific implementation of the blinding method, which posed risk of serious bias; Patients may be heterogeneous due to inconsistent characteristics such as age and course of disease among studies; Total effective rate included a small number of samples so that the results may be inaccurate; There is statistical heterogeneity among the MMSE score groups. The GRADE evidence assessment showed that all the obtained evidence quality ratings were low to very low. The evaluation results are shown in Table 2.

**Table 2:** GRADE evidence.

Quality` assessment							No of patients		Effect		Quality	Importance
No of studies	Design	Risk of bias	Inconsistency	Indirectness	Imprecision	Other considerations	Gunao-Yizhi decoction combined with donepezil	Donepezil	Relative (95% CI)	Absolute		
**Clinical effectiveness**												
6	Randomized trials	Serious*	Serious†	No serious indirectness	Serious‡	None	209/240 (87.1%)	149/239 (62.3%)	RR 1.4 (1.25–1.56)	249 more per 1000 (from 156 more to 349 more)	⊕ΟΟΟ Very low	Critical
								59.4%		238 more per 1000 (from 149 more to 333 more)		
**MMSE score (better indicated by lower values**)												
9	Randomized trials	Serious*	Serious†	No serious indirectness	Serious§	None	422	417	-	MD 3.12 higher (2.45–3.78 higher)	⊕ΟΟΟ Very low	Critical
**HDS score (better indicated by lower values**)												
9	Randomized trials	Serious*	Serious†	No serious indirectness	No serious imprecision	None	414	408	-	MD 3.62 higher (3.11–4.13 higher)	⊕⊕ΟΟ Low	Important
**Serum SOD level (better indicated by lower values**)												
6	Randomized trials	Serious*	Serious†	No serious indirectness	No serious imprecision	None	287	282	-	MD 11.73 higher (9.96–13.5 higher)	⊕⊕ΟΟ Low	Important
**Serum MDA level (better indicated by lower values**)												
6	Randomized trials	Serious*	Serious†	No serious indirectness	No serious imprecision	None	287	282	-	MD 1.13 lower (1.34–0.93 lower)	⊕⊕ΟΟ Low	Important

* All the studies did not mention the blind method or the specific implementation of the blind method, which has a risk of serious bias.

† Patients may be heterogeneous due to inconsistent characteristics such as age and course of disease among studies.

‡ This group included a small number of samples so that the results may be inaccurate.

§ There was statistical heterogeneity among the MMSE score group.

HDS = Hasegawa dementia scale, MDA = malonaldehyde dismutase, MMSE = mini-mental status examination, SOD = superoxide dismutase.

## 4. Discussion

The etiology of vascular dementia is complex. Vascular dementia belongs to the category of “Dementia” and “Madness” in traditional Chinese medicine. Its manifestations are “Forgetfulness” and “Madness”, which have been recorded in *Inner Canon of the Yellow Emperor*. Deficiency and damage of all the 5 internal organs can lead to vascular dementia. Still, kidney deficiency can lead to brain impotence and tissue reduction because the kidney governs the bone and produces marrow.^[20]^ Because the kidney regulates the metabolism of water and fluid, when the storage of water and fluid ceases, phlegm turbidity develops, leading to dementia. As a result, despite the fact that the disease is located in the brain, VaD is most closely associated with kidney deficiency and phlegm turbidity. To treat vascular dementia, modern doctors begin by tonifying the kidney, filling essence, resolving phlegm, and opening orifices. In the prescription of Gunao-Yizhi decoction, rehmannia glutinosa, lycium barbarum, polygoni multiflori preparata, ligustrum lucidum are monarch drugs, which are beneficial to filling marrow and nourishing kidney; rhodiola rosea, semen ziziphi spinosae, and polygala tenuifolia nourish the heart and intelligence; acorus tatarinowii schott and lumbricus eliminate phlegm and open orifices; epimedium brevicornum and semen astragali complanati strengthen the kidney and benefit essence; codonopsis pilosula and pueraria lobata nourish yin and generate fluid. Combined with these herbs, they play the effects of strengthening the kidney, benefiting essence, removing phlegm, and resolving turbidity. Liang Mingzhan et al^[21]^ proved through network pharmacological research that the drug combination of rehmannia glutinosa-radix polygoni multiflori preparata-acorus tatarinowii schott-rhizoma Chuanxiong regulates the pathways related to neurodegenerative diseases and inflammatory vascular diseases acts on the endocrine system and nervous system and plays a role in the treatment of VaD through the way of tonifying the kidney and with the help of key targets such as APP, TNF, and JUN. Modern pharmacological studies have confirmed that salidroside extracted from rhodiola ppcan significantly improve the cognitive impairment of 2-VO rats and increase the level of long-term potentiation in the hippocampus to treat VaD;^[22]^ The ethanol extract of fructus corni has antioxidant activity and protective effect of antioxidant enzyme system;^[23]^ The ethanol extract of semen astragali complanati has strong reducibility and antioxidant activity;^[24]^ codonopsis pilosula extract has the function of improving learning and memory.^[25]^

According to the findings of this study, Gunao-Yizhi decoction combined with donepezil was more effective than donepezil alone in terms of clinical efficacy. Gunao-Yizhi decoction combined with donepezil is more effective than donepezil alone in improving MMSE scores. The combination of Gunao-Yizhi decoction and donepezil can improve HDS scores more than donepezil alone. Gunao-Yizhi decoction combined with donepezil is more effective than donepezil alone in improving serum SOD levels; in lowering serum MDA levels, Gunao-Yizhi decoction combined with donepezil is more effective than donepezil alone. Based on the above results, it can be concluded that Gunao-yizhi decoction combined with donepezil is superior to donepezil alone in terms of improving cognitive function, increasing intelligence, and enhancing the anti-aging ability of the nervous system in patients with VaD. At the same time, this study also has some limitations. The literature included in this study is all Chinese literature published in domestic journals; The sample size of the included studies is small, and the impact of accidental events on the outcome cannot be excluded; There is a certain risk of bias in the included literature, which is embodied in random method, distribution concealment, blinding method implementation, outcome index, and so on; only 1 study reported the adverse reactions after medication, so the safety research was not comprehensive enough; because only 1 study reported adverse reactions after medication, the safety research was insufficiently comprehensive. All of the GRADE evidence ratings are low or very low, implying that a larger sample size, stricter implementation of the distribution concealment and blinding method, and evaluation and demonstration of randomized controlled trials with more detailed records of adverse reactions are required to better guide clinical application.

## 5. Conclusion

To sum up, this study shows that combined therapy using Gunao-Yizhi decoction and donepezil had better effects on VaD than donepezil alone only in terms of total effective rate, MMSE score, HDS score, serum SOD level and serum MDA level.

As a result, combining Gunao-Yizhi decoction with donepezil can provide a certain reference value for clinical practice. However, larger sample size multi-center randomized controlled trials are required to provide a more reliable basis for its clinical application.

## Author contributions

**Conceptualization:** Yibin Hu, Lijuan Zhang, Kangfeng Wang.

**Data curation:** Lijuan Zhang, Xiuju Guan, Changning Liu.

**Formal analysis:** Xiuju Guan, Hanru Hou, Shuyue Bi, Mingxiang Li.

**Methodology:** Lijuan Zhang, Shuyue Bi, Changning Liu, Mingxiang Li.

**Software:** Xiuju Guan, Hanru Hou, Shuyue Bi.

**Writing – original draft:** Yibin Hu.

**Writing – review & editing:** Kangfeng Wang.
